# Vitamin D Synthesis Following a Single Bout of Sun Exposure in Older and Younger Men and Women

**DOI:** 10.3390/nu12082237

**Published:** 2020-07-27

**Authors:** Jenna R. Chalcraft, Linda M. Cardinal, Perry J. Wechsler, Bruce W. Hollis, Kenneth G. Gerow, Brenda M. Alexander, Jill F. Keith, D. Enette Larson-Meyer

**Affiliations:** 1 Department of Family and Consumer Sciences, University of Wyoming, Laramie, WY 82071, USA; jchalcra@uwyo.edu (J.R.C.); jkeith5@uwyo.edu (J.F.K.); 2 Billings Clinic, Cody, WY 82414, USA; lcardinal06@gmail.com; 3 Alpenglow Instruments, Laramie, WY 82072, USA; perrywechsler@alpenglowinstruments.com; 4 Dr Bruce Hollis’ Laboratory, Medical University of South Carolina, Charleston, SC 29425, USA; hollisb@musc.edu; 5 Department of Mathematics & Statistics, University of Wyoming, Laramie WY 82071, USA; gerow@uwyo.edu; 6 Department of Animal Sciences, University of Wyoming, Laramie, WY 82071, USA; balex@uwyo.edu; 7 Department of Human Nutrition, Foods & Exercise, Virginia Tech, Blacksburg, VA 24061, USA

**Keywords:** ageing, older persons, sensible sun exposure, cutaneous synthesis, vitamin D_3_, natural sunlight, serum 25(OH)D

## Abstract

Older adults are frequently cited as an at-risk population for vitamin D deficiency that may in part be due to decreased cutaneous synthesis, a potentially important source of cholecalciferol (vitamin D_3_). Previous studies found that cutaneous D_3_ production declines with age; however, most studies have been conducted ex vivo or in the photobiology lab. The purpose of this study was to characterize the response of vitamin D metabolites following a 30-min bout of sun exposure (15-min each to the dorsal and ventral sides) at close to solar noon in younger and older adults. Methods: 30 healthy individuals with skin type II/III were recruited; a younger cohort, aged 20–37 (*n* = 18) and an older cohort (*n* = 12), age 51–69 years. Exposure was at outer limits of sensible sun exposure designed to enhance vitamin D synthesis without increasing risk of photo ageing and non-melanoma skin cancer. Serum D_3_ concentration was measured at baseline, 24, 48 and 72 h post-exposure. Serum 25(OH)D was measured at baseline and 72 h post-exposure plus 168 h post-exposure in the older cohort. Results: D_3_ increased in response to sun exposure (time effect; *p* = 0.002) with a trend for a difference in D_3_ between cohorts (time*group; *p* = 0.09). By regression modeling of continuous data, age accounted for 20% of the variation in D_3_ production. D_3_ production decreased by 13% per decade. Despite changes in D_3_, however, serum 25(OH)D did not change from baseline to 72 or 168 h post exposure (*p* > 0.10). Conclusions: Serum D_3_ concentration increased significantly in response to outdoor sun exposure in younger and older adults. While ageing may dampen cutaneous synthesis, sunlight exposure is still a significant source of vitamin D_3_.

## 1. Introduction

Accumulating evidence has documented that vitamin D deficiency is associated with the onset and progression of a variety of chronic diseases including cardiovascular disease, type 2 diabetes, immune system diseases, neuropsychiatric disorders and certain cancers [1]. Older adults are frequently cited as an at-risk population for vitamin D deficiency [2,3,4]. Population-based studies suggest a high prevalence of vitamin D deficiency (serum 25(OH)D <20 ng/mL) among older adults (>60 to 65 years) that varies by location and ethnicity, and ranges from 12.1% of individuals with mixed ancestry in Greater Toronto (43° N) [5] to 37.3% in Mexico (<32° N) [6] and 45% in the Netherlands (52° N) [7]. Recent analysis of data from the National Health and Nutrition Examination Survey (NHANES 2011–2014), however, found that while vitamin D status differed by age, the prevalence of at risk of deficiency or inadequacy (defined as serum 25(OH)D <20 and 50 nmol/L, respectively) was highest in adults aged 20–39 years (7.6 and 23.8%) compared to adults ages 40–59 years (5.7 and 18.6) and those 60 years or older (2.9 and 12.3) [8], with no differences between men and women. Indeed, all adults and children may be at risk for vitamin D deficiency, especially when living in high latitude regions (>35°) [9] or receiving limited sun exposure. Nonetheless, deficiency in older adults is most often a combination of age-related changes in vitamin D metabolism and lifestyle factors. Age-related alterations in vitamin D metabolism include decreased epidermal 7-dehydrocholesterol (DHC) concentration, reduced thickness of the epidermis [10,11], reduced dermal [12] (and epidermal) vitamin D production, increased adiposity [13] (fat sequestration), decreased renal 1,25(OH)_2_D synthesis [14,15,16] and increased 1,25(OH)_2_D catabolism [16]. The reduced dermal capacity to synthesize vitamin D at age 65 for example has been estimated to be ~25% of that of a 20–30-year-old exposed to the same amount of radiation [12,17]. Lifestyle factors associated with deficiency include poor appetite, low vitamin D intake, limited sun exposure/sun avoidance, increased clothing coverage/sunscreen use, reduced physical activity and financial constrains [3,9,14,18,19,20].

A number of studies in older adults, on the other hand, have demonstrated that exposure to ultraviolet B (UVB, 290–315 nm) radiation is an effective strategy for increasing serum 25(OH)D concentration [21,22,23,24]; this may suggest that sun avoidance rather than reduced synthesis per se is an important risk factor for suboptimal status in older individuals. Most of the aforementioned studies, except for that of Reid et al. [24], however, have been conducted in the photobiology lab following delivery of a measured dose of UVB. Further research using ambient sun exposure is necessary to continue to characterize the pattern of cutaneous vitamin D production in older versus younger adults in a natural environment (i.e., the sun is freely available) and determine the effectiveness of sun exposure therapy on vitamin D status.

Despite its potential benefit, however, sunlight exposure is considered a controversial way to maintain vitamin D status [25]. Many health authorities including the World Health Organization [26], the American Cancer Society [27] and the surgeon general [28] emphasize sun abstention, especially during mid-day when the sun’s ultra-violet (UV) rays are the most potent. UV damage from too much sun exposure plays a role in the development of skin cancer and is considered a public health risk [25]. General public health efforts target sunscreen use, skin coverage with clothing and/or a sun hat and sun avoidance to reduce exposure to UVA and UVB radiation [3] and prevent cancer and photo ageing. While these public health efforts are understandable from the viewpoint of prevention of nonmelanoma skin cancer, they neglect the potential physiological benefits of mindful sunlight exposure that includes elevation of vitamin D status [25] and the potential influence of sun exposure and vitamin D on health in ageing individuals [29]. “Sensible sun exposure” is the practice of obtaining the minimum sun exposure required for adequate vitamin D synthesis followed by application of sunscreen or clothing coverage; and is often characterized as sun exposure of 5–30 min, two–three times per week to the arms, legs and torso during 10:00 h to 15:00 h [2,30]. The specific duration required within this time frame is dependent on skin type, previous tanning, latitude, season and environmental conditions. The minimal erythemal dose (MED), or the minimum quantity of UVB that induces a slight erythema 16–24 h post exposure, is a common dosage employed for sensible sun exposure guidelines [31]. The standard erythemal dose (SED) is a standardized method for quantifying erythemal UV radiation (UVR) dose and is becoming more commonly used because the MED varies from person to person even within the same skin type. The SED is defined as 100 J/m^2^ [32]. For adults with a fair complexion, one MED is equal to about 10–12 min of full body exposure during peak summer sun for all skin types [33]. One MED may be considered equivalent to ~2.5 to 3.5 SEDs depending on skin type [34].

As “sensible sun exposure” may be a simple, cost effective method to obtain optimal 25(OH)D concentration and/or prevent deficiency in older populations, the purpose of this study was to characterize the response of vitamin D_3_ and 25(OH)D to a single bout of outdoor sun exposure of 15 min to both the dorsal and ventral sides of the body (30 min total) in older and younger adults. We hypothesized, based on previous literature, that a single bout of sun exposure would be effective at increasing serum vitamin D_3_ concentration but that older individuals would demonstrate a reduced response to natural sunlight exposure compared to younger individuals.

## 2. Materials and Methods

This study consisted of exposing younger (19–39 years) and older (51–69 years) adults to a 30-min bout of natural outdoor sunlight (15 min each on the dorsal and frontal sides of the body) at moderate-altitude (2194 m, 41.3° N) in late spring/early summer (close to the summer solstice). Both research procedures were reviewed and approved by the Institutional Review Board at the University of Wyoming (Protocols # 20150317EL 00717 and #20180219EL01882) and approved in April 2015 and April 2018, respectively. Volunteers provided written, informed consent before participation.

### 2.1. Participants

Young male and female volunteers (19–39 years of age) and healthy community-dwelling older adults (50–70 years) with skin type II or III were recruited from the local community in the spring (April–June) of 2015 (younger cohort) and spring of 2018 (older cohort) through advertisements posted on a university campus and in the local community. To be eligible, participants had to have skin type II or III and must not have a personal or family history of skin cancer or melanoma. Skin type was self-identified using the Fitzpatrick skin typing scale [35], which was read to potential participants. Those who self-identified as skin types II (white, fair; blond or red hair; blue, green or hazel eyes; usually burns, tans minimally) or III (cream white, fair with any hair or eye color; sometimes mild burn, tans uniformly) were permitted into the study. Exclusion criterion included: current vitamin D supplementation or supplementation one month before the study, current use of medications that have the potential to alter vitamin D status or photosensitivity, travel within three months to a sunny location close to the equator, appearing visibly tanned or the inability to either refrain from sunlight or artificial UV exposure from screening until the scheduled sun exposure session and for 3 days following this session or fully participate in the study due to work, school or personal scheduling constrains.

A total of 51 volunteers in the younger-aged cohort and 45 in the older-aged cohort responded to the advertisement. Interested volunteers were initially screened by phone and asked a series of questions about their general health, personal and family risk of skin cancer, use of supplements and perceived skin type. In the younger age cohort, five were unable to complete screening and 15 were unable to participate due to schedule conflicts. Thirteen were deemed ineligible for participation because they reported a family history of skin cancer, were taking vitamin D supplements, did not meet skin type guidelines or had travelled to a sunny location and/or spent a generous time outside without sunscreen in the previous three months. In the older-aged cohort, the majority of interested participants were not eligible due to either a personal or family history of melanoma or pre-cancerous skin lesions. Additional reasons for ineligibility included skin type, tanning bed use, recent travel to a sunny location or use of medications that might influence vitamin D status. Of the 51 and 45 individuals interviewed in the younger and older-age groups, 18 and 12 were both eligible and available to participate in the study.

### 2.2. Baseline Measurements

Baseline anthropometric measurements, which included height, weight, and body composition measured by dual energy x-ray absorptiometry (DXA) (Lunar Prodigy, GE Healthcare, Fairfield, CT, USA), were obtained on all participants. Height and weight were measured without shoes and in minimal clothing using a standing digital scale (Tanita, Tokyo, Japan) and stadiometer (Invicta Plastics, Leicester, England). Body mass index (BMI) was calculated as weight divided by height in meters squared and body surface area (BSA) was calculated using the Mosteller equation [36]. Participants also completed a vitamin D-specific food frequency and lifestyle questionnaire (FFLQ) [37]. The FFLQ addresses questions about the frequency of consumption of vitamin D-containing foods, vitamin D supplementation, sun exposure and tanning bed usage.

### 2.3. Sunlight Exposure

Following the baseline visit, volunteers were scheduled to participate in one of several sunlight exposure sessions or test days. The exposure days had to meet certain criteria (cloudless sunny day with a minimum ambient temperature of 16 °C and a maximum of 32 °C) and were selected based on weather patterns, the university calendar and participant/research staff availability for the exposure day and for the 72 h (all participants) to 168 h period (older group only) following exposure. The period of April through June was selected to ensure that exposure occurred to early-season naive skin (i.e., skin that had not been recently tanned). On each test day, a sun exposure station was set up in a mostly sheltered outdoor environment at a local park (younger group) or landscaped courtyard (older group). Volunteers had pre-sun exposure (baseline) blood drawn for analysis of serum vitamin D_3_ (cholecalciferol), vitamin D_2_ (ergocalciferol) and 25-hydroxyvitamin D (25(OH)D) immediately before undergoing sunlight exposure that consisted of exactly 15 min of exposure to both the front and back sides of the body (30 min total) between 11:30 and 13:00 h standard time (12:30 to 14:00 h daylight saving time) while lying supine and prone, respectively, in shorts (men) or shorts and sports bra (women). The shorts worn had a 2- to 3-inch inseam or were cuffed to a similar length. This amount of skin exposure was estimated to be approximately 43% of BSA for men and 41% for women. A member of the research team closely monitored the sessions to ensure compliance with time and protocol and to watch for signs of notable erythema. Close to solar noon was selected because UVB is most intense during this time, which allowed for more efficient vitamin D synthesis and reduced risk for skin cancer [38,39]. Sunscreen (Equate Broad Spectrum SPF, Bentonville, AR) was applied to the face and sunglasses were provided to all participants who did not bring their own protective eyewear. Following carefully timed exposure, volunteers took immediate cover in a designated area indoors and were served a light lunch. Participants were asked to completely avoid outdoor sunlight for the next 72 h; if outdoor daytime exposure was unavoidable, participants were asked to cover up with full-body clothing and/or the provided sunscreen. 72 h was extended to 168 h in the older participants. To prevent the chance that some newly synthesized vitamin D was sloughed off with skin cells [40], volunteers were asked not to bath, shower or swim until after their 24-h blood draw.

### 2.4. Blood Draws and Analyses

Blood was drawn at baseline and approximately 24-, 48- and 72-h after initial exposure for the analysis of serum vitamin D_3_, vitamin D_2_ and 25(OH)D concentration. Blood was also obtained at 168 h post-exposure in the older group. Blood was allowed to coagulate at room temperature for 30-min and then centrifuged at 5800 rpm for 15 min (VWR Clinical 100, VWR International, Woodbridge, NJ). Serum was frozen until analysis. Vitamin D_3_/D_2_ was analyzed via liquid chromatography-mass spectrometry (LC/MS) by a commercial laboratory (Heartland Assays, Ames, IA). The intra- and inter assay coefficient for these assays is <3% and <6%, respectively. 25(OH)D concentration was analyzed via Diasorin 25(OH)D RIA (B.W. Hollis, Charleston, SC). The intra- and inter-assay coefficient for 25(OH) D assay is less than 10%.

### 2.5. Estimates of UV-Irradiance

UV irradiance was measured during sun exposure using a meteorological grade UV instrument (Total Ultraviolet Radiometer model 27901, The Eppley Laboratory, Inc. Newport, RI) and a logging data acquisition system (National Instruments, Austin, TX). UV data were measured in one-second intervals for all exposure days in the younger participants and for a single representative day in the older participants due to computer malfunction. Total UV exposure was calculated per participant from actual recorded start and stop times on the test day or the representative test day. Data from the Eppley radiometer is recorded in native units of volts and were then converted to irradiances using the device calibration coefficient of 1 mv = 2.28 mW/cm^2^. The UVA/UVB ratio of 20:1 or 5% of the total UV irradiance was used to estimate UVB [41]. The sum of irradiances for 30 min (1800 s) were then calculated and subsequently converted to a standard erythemal dose (SED) using the conversion: 1 SED = 100 J/m^2^ [32]. As participants were supine and prone during exposure, total exposure was divided by two to represent the exposure to each body side. Prior to data collection, baseline data (or calibration data) were measured by taking running averages of one-minute data segments with the radiometer shielded from any incident light; these data indicated that there was no significant offset to the total UV data collected.

### 2.6. Statistical Procedures

Statistical analyses were performed using IBM SPSS version 26.0 (Chicago, IL, USA) and Minitab version 19.0 (State College, Pennsylvania, USA). Independent Samples t-tests were used to test for differences between older and younger groups for baseline physical characteristics, baseline serum vitamin D metabolites, and sun exposure variables. To address our primary aim, Repeated Measures Analysis of Variance (ANOVA) was initially used to evaluate the effect of group (older vs. younger) by time (baseline, 24, 48 and 72 h) on serum D_3_ concentration following sun exposure. Paired T-Tests with Bonferroni correction were then used post hoc to test for differences in serum vitamin D_3_ from baseline to 24, 48 and 72 h, and to test for differences between serum 25(OH)D at baseline and 72 h (and baseline and 168 in the older group). A Fishers Exact Test was used to evaluate differences in timing of the peak serum D_3_ concentration following sun exposure. ANOVA was also employed to evaluate sun exposure and UVB irradiance variables between and within studies on peak vitamin D_3_ concentration following sun exposure including sun exposure day/year, temperature during sun exposure and estimated UVB dose (SED). The best subsets method was utilized to identify predictors of D_3_ production from baseline to peak concentration that included important inter-subject characteristics and confounders (age, baseline serum vitamin D_3_ concentration, fat mass, lean mass, reported baseline vitamin D intake and sun exposure history). A simple linear regression model for D_3_ production was subsequently constructed to evaluate the relationship between age and D_3_ production. Log transformation was performed to best quantify the relative relationship.

## 3. Results

### 3.1. Subject Characteristics

All participants (*n* = 30) were Caucasian who identified with having skin type II or III. The characteristics of the participants, including estimated vitamin D intake and reported sun exposure are summarized in Table 1. The older group was significantly shorter than the younger group but no other differences (other than age) were observed. There were no differences in average daily vitamin D intake over the past 3 months and average daily sun exposure between the older and younger groups (Table 1).

### 3.2. Sun Exposure and Estimates of UVB Irradiance

Weather conditions and participant availability allowed for three mostly cloudless, sun exposure days in the spring of 2015 and four similar days in the Spring of 2018. In 2015, days were 68, 19 and 4 days prior to the summer solstice (21 June) and had recorded temperatures (typically at 12:53 h) of 19.4°, 25° and 23.3 °C, respectively. In 2018, days were 35, 29, 17 and 9 days prior to the summer solstice, with recorded temperatures of 21.6°, 21.1°, 25.6° and 25.6 °C, respectively. Average body surface area exposed was estimated to be 0.78 m^2^ ± 0.02 in the younger cohort and 0.73 m^2^ ± 0.03 in the older cohort and average UVB radiant exposure estimated via the radiometer was 386.3 ± 9.2 mJ/cm^2^ and the SED was 38.6 ± 0.92 for whole body (19.3 for the frontal and dorsal sides) for the younger group and was 377.3 ± 1.6 mJ/cm^2^ with an average total SED of 37.7 ± 0.16 (18.85 for the frontal and dorsal sides). Neither BSA exposed nor SED differed between groups (*p* > 0.10).

### 3.3. Impact of Sun Exposure on Serum D_3_ and 25(OH)D

**Figure 1:**
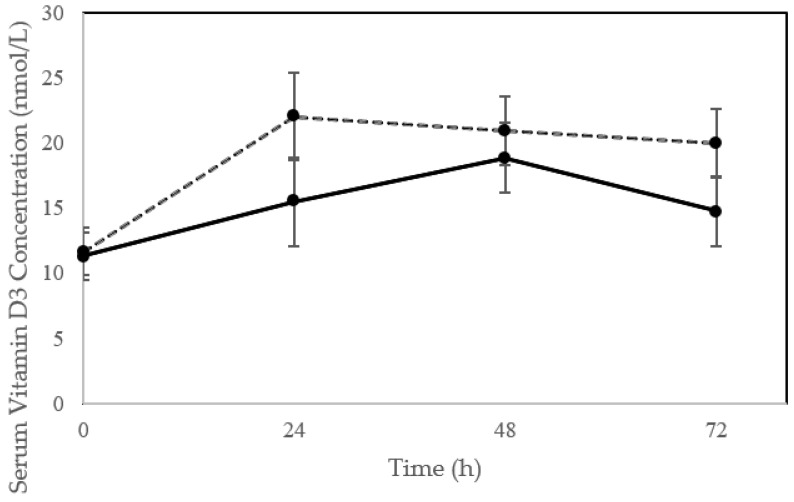
Vitamin D_3_ concentration following a single bout of sun exposure (time 0) in older (solid line) compared to younger (dashed lines) adults. Serum vitamin D_3_ concentration increased significantly post exposure (time effect; *p* < 0.002) with a trend for a difference in vitamin D_3_ response between older and younger individuals (time*group; *p* = 0.09). Error bars represent ± SE.

In the 24 h following sunlight exposure, only one participant (younger cohort) had observable signs of erythema. Following the single bout of sun exposure, serum vitamin D_3_ concentration increased significantly in the 24 to 48 h post exposure (time effect; *p* < 0.002) with a trend for a difference in vitamin D_3_ response between older and younger individuals (time*group; *p* = 0.09) and no group effect (*p* = 0.21) (Figure 1). The average increase was of 76.4 ± 83.7%, 88.1% ± 73.3 and 54.1 ± 52.5% at 24, 48 and 72 h, respectively, which were significantly greater than baseline at all points (*p* < 0.017). Peak concentration in serum D_3_ were generally observed between 24 and 48 h with a few participants, all in the younger cohort, experiencing slight upward trends in D_3_ concentration from 48 to 72 h. Overall there was a trend for older individuals to peak later (at 48 h) compared to younger individuals (at 24 h) (*p* = 0.0875), with one participant in the older cohort experiencing a peak at 24 h and nine at 48 h compared to seven participants in the younger cohort who peaked at 24 h and eight between 48 and 72 h. Two subjects in the older and three in the younger cohorts (16.7%) did not experience an increase in serum D_3_ at any time point. The average increase in D_3_ at peak was 9.8 ± 7.8 nmol/L (including the non-responders) and was not different between cohorts (7.7 ± 5.7 vs. 11.2 ± 8.8 nmol/L, older vs. younger, *p* = 0.25).

**Figure 2:**
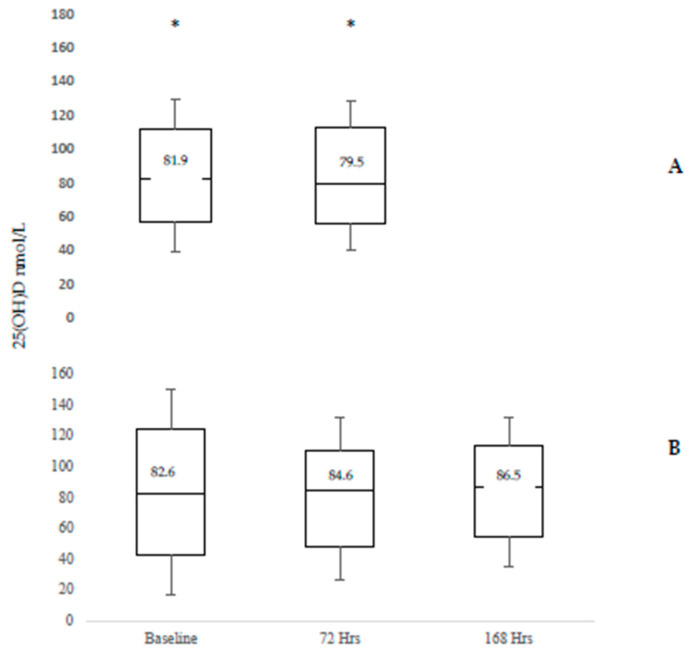
25(OH)D Concentration at baseline, 72 h and 168 h post exposure to natural sunlight for a total of 30 min in the Younger (Panel A) and Older (Panel B) Adult Cohorts. * represents single data points that were greater than three times the interquartile range.

Despite changes in serum vitamin D_3_ concentration, serum 25(OH)D concentration did not change following sunlight exposure and was not different between baseline and 72 h (*p* = 0.561) or baseline and 168 h post exposure (*p* = 0.237) (Figure 2).

### 3.4. Predictors of D_3_ Production

Using the best subsets method with the log of D_3_ production (change from baseline to peak) as the dependent variable and age, baseline serum D_3_ concentration, fat mass, lean mass, reported vitamin D intake and reported sun exposure history entered as independent variables, the single best predictor of D_3_ production was age (*r^2^* = 0.238). The best pairs of predictors were age and lean body mass (*r^2^* = 0.30) and age and baseline serum D_3_ concentration (*r^2^* = 0.298). Age, baseline serum D_3_ concentration and lean body mass were the best triplet of predictors (*r^2^* = 0.352). Fat mass, reported time spent outside and vitamin D intake were not selected as predictors until four, five or six predictors were entered, and contributed little to the model.

### 3.5. Modeling of D_3_ Production with Ageing

**Figure 3:**
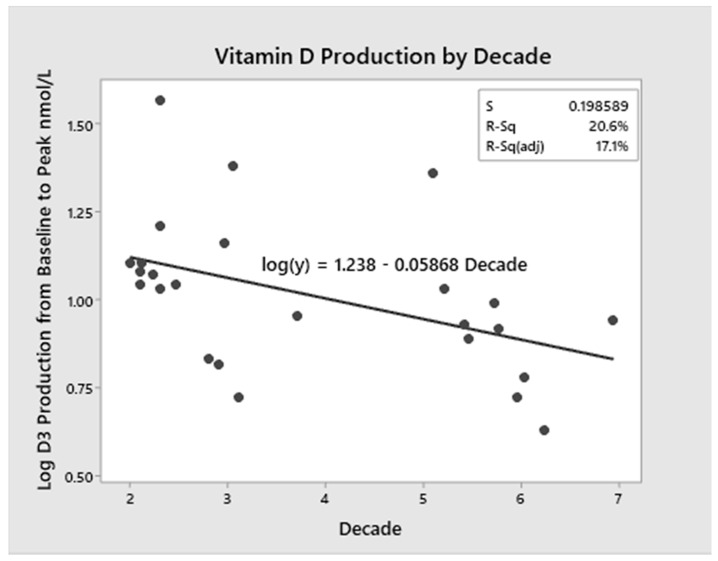
Vitamin D_3_ Production and Age Model. The linear regression model with age as the independent variable and log D_3_ production was constructed. Age accounted for 20% of the variance in D_3_ production (*r^2^* = 0.206).

**Figure 4:**
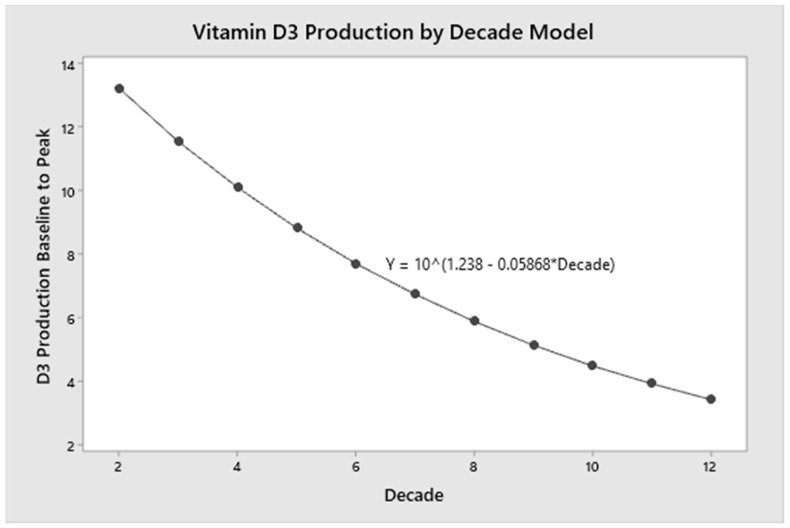
Vitamin D_3_ Production Age Continuum Modeling. The simple linear regression model with decade as the independent variable and log D_3_ production was used to demonstrate the 13% decrease in D_3_ production per decade of life. D_3_ production at age 70 years is approximately half that produced at age 20. The graph demonstrates that D_3_ production is possible even in the later decades of life.

As shown in Figure 3, a linear regression model using data from both cohorts across the age spectrum (21 to 69 years) was created with age as the independent variable and log D_3_ production (change from baseline to peak) as the dependent variable (*p* = 0.023). Age accounted for 20 percent of the variance in D_3_ production (*r^2^* = 0.206). The regression model (Figure 4) further demonstrated that for every decade of life, there is a 13 percent decrease in mean D_3_ production.

## 4. Discussion

The primary aim of the current study was to characterize the response of a single bout of sun exposure on cutaneous vitamin D synthesis in a cohort of younger and older adults, and determine if the response in older adults differed from that of a younger cohort. Overall, we found that 30 min of sun exposure (15-min to the arms, torso and legs on both the front and backsides of the body while lying in the supine and prone positions) significantly increased serum vitamin D_3_ by an average of 9.8 nmol/L with no significant differences in cohorts. The peak response in D_3_ concentration to a single bout of “sensible sun exposure,” however, was about 1/5th the response observed following delivery of 1 MED in a photobiology laboratory [42]. A model created from continuous data on peak D_3_ concentration in response to sun exposure found evidence that D_3_ production in adults declines with ageing (by ~13% per decade) but is still possible at 120 years of age. To our knowledge, this is the first study to evaluate the effect of a single bout of sensible sun exposure on vitamin D_3_ concentration using natural sunlight exposure of a carefully monitored duration as the sole UVB source, which is important for understanding the implication of “sensible sun exposure” guidelines in older as well as younger individuals.

Previous calculations from studies performed in a photobiology laboratory suggest that the equivalent 10,000–25,000 IUs of vitamin D can be synthesized from UVB irradiation in individuals wearing a bikini in peak July sun [43] after exposure of 1 MED, suggesting that sun exposure is the most significant source of vitamin D. While sufficient exposure to the UVB radiation from sunlight is important for cutaneous vitamin D synthesis, too much sun exposure increases the risk for photo ageing and skin cancer [33]. Guidelines for “sensible sun exposure,” which are thought to promote vitamin D synthesis at minimal risk of excess exposure, were established from studies conducted within a photobiology laboratory. Only a few studies have investigated and compared (rather than modeled) the efficacy of solar and artificial UVB radiation on cutaneous synthesis [44] or estimated the outdoor exposure time necessary to achieve a serum 25(OH)D concentration equivalent to a specific oral dose (e.g., 1000 IU) of supplemental vitamin D according to season, location and skin type [45].

The most accepted guidelines established by Holick et al. [2] indicate that exposing the arms, hands and face to one-third to one-half of a MED, which is about five minutes at noon in Boston for individuals with skin type II, with a frequency of two to three times per week during the spring, summer or fall, is more than adequate to achieve sufficient vitamin D status [46]. More specific guidelines are difficult to make because of the multiple variables involved in cutaneous production of vitamin D, such as season, latitude, cloud cover, skin pigmentation and body surface area exposed. Updated guidelines suggest exposure of the arms, legs and torso (when possible) to sunlight for approximately 25% to 50% of the time it would take to develop a mild sunburn (e.g., 1 MED) for this same frequency (two–three times/week), and exemplify that if 30 min of noontime sun would cause a mild sunburn, than 10 to 15 min of exposure (followed by sun protection) should be sufficient for adequate vitamin D synthesis [30]. The present study suggests that a single session of solar exposure to both the front and back-sides of the body at the outer limits of the “sensible sun exposure” guidelines was sufficient to promote D_3_ synthesis in most individuals aged 20 to 69 years who self-identified with skin types II and III, but also that some individuals may be non-responsive to a single exposure. This is of interest because UVB exposure achieved in a natural environment is likely to be more variable than that of a photobiology laboratory, where the dose of UVB delivered can be controlled and directly quantified. For example, one study at a latitude of 56° N concluded that artificial UVB exposure to the hands and face was at least eight times as effective at increasing 25(OH)D synthesis than solar UVR from early spring exposure under natural conditions [44]. Another estimated from spectral characteristics that sunbeds were ~25–30% more efficient in producing pre-vitamin D than mid-June sun at 59° N latitude [34]. Despite the variability of natural sunlight, achieving vitamin D from “sensible sun exposure” is inexpensive and freely available.

Results of the current study provide important data to help address the influence of ageing on vitamin D synthesis and the appropriate sensible sun exposure guidelines for older individuals. Previous research has demonstrated decreased cutaneous D_3_ production in older adults. A classic ex vivo study of MacLaughlin and Holick found that skin samples obtained from older adults aged 77 to 82 had significantly less 7-DHC concentration compared to skin samples from young adults [12]. A two-fold decrease in pre-vitamin D_3_ synthesis in skin samples from the older adults compared to those of an 8- and 18-year-old was also observed [12]. Another study in the photobiology laboratory found that older adults ages 62–80 years with type III skin produced three times less D_3_ than young adults age 20–30 years with this same skin type following simulated whole-body sunlight exposure of 32 mJ/cm^2^ [17]. To our knowledge, however, previous research has not firmly established a specific age at which a reduction in cutaneous D_3_ occurs.

In the current study, both older and younger individuals experienced significant positive increases in circulating D_3_ following sun exposure, with only a trend for a different pattern of response between cohorts. The older cohort tended to experience a peak in circulating D_3_ at 48 h in contrast to the younger cohort, who experienced peak D_3_ concentration nearly equally at 24 h or 48 h. While studies in the photobiology laboratory indicated that D_3_ concentrations typically peak within 24 to 48 h following UVB exposure [42], the later peak time in the majority of the older cohort (nine out of 10 participants) could be explained by an age-related decline in the surface area between the dermis and epidermis that ultimately affects nutrient exchange [47,48]. Reduction in basal cell growth in keratinocytes is a major consequence of epidermal thinning [47,48], which has the potential to influence D_3_ production.

Perhaps more importantly, our regression modeling conducted throughout the adult age continuum (ages 20–69) showed that alterations in cutaneous vitamin D_3_ production declines throughout adulthood. Our regression modeling (Figure 3 and Figure 4) demonstrated that for every decade of life, there is a 13 percent decrease in D_3_ production (1.3% per year); by the seventh decade, vitamin D_3_ production is approximately half that at age 20. The model, however, demonstrates that even at 120 years of age, vitamin D_3_ production is still possible. This is in support of thinking that “…skin has a great capacity to make vitamin D even in the elderly” [2]. This also highlights that there is no specific age at which cutaneous synthesis suddenly stops but rather that it declines slowly over the years.

While not an original intent of the study, the current study also provides interesting results concerning “non-responders” that constituted approximately 17 percent of both cohorts. These non-responders did not have an increase in circulating vitamin D_3_ concentration following a full 30-min of exposure. Curiously, however, there were little differences between these five individuals and the rest of the group other than their baseline serum D_3_ concentration, which were on average higher than the 25 responders (34 vs. 11 nmol/L) despite reporting both limited sun exposure and no supplemental vitamin D for at least 3 months prior to study initiation. One individual in the older cohort was also the largest participant, with Class II obesity (BMI > 35.2 kg/m^2^; body fat = 43.4%). These data combined with our regression models (best subset method) suggest that baseline vitamin D status may influence cutaneous vitamin D synthesis or at least its appearance in circulation. Biochemically it is well-recognized that vitamin D synthesis is regulated through control mechanisms that prohibit vitamin D intoxication through sun exposure by converting excess pre-vitamin D_3_ to biologically inert molecules including tachysterol and lumisterol [33]. Baseline 25(OH)D concentration, which averaged 33.1 ± 3.0 ng/mL and were generally sufficient, could also have significantly impacted cutaneous vitamin D synthesis [32] even though D3 was more directly influential in the modeling. Other possible explanations include genetic variation [49] or the misclassification of skin type [45] in the non-responders. For example, genetic variants in close proximity genes involved in cholesterol synthesis, hydroxylation and vitamin D transport have been linked to the elevated risk of vitamin D insufficiency in individuals of European descent, and could be present in some of our non-responders [49]. Alternately, misclassification of individuals with skin types IV and V may have resulted in decreased D_3_ synthesis because time of exposure was not sufficient in these skin types [32].

Additionally, while D_3_ increased significantly in response to the sun exposure session, 25(OH)D did not change from baseline to 72 h post exposure in the younger and older cohorts or from baseline to 168 h in the older cohort who had data collected at this additional time point. The observed phenomenon may be a result of a combination of factors including the single exposure, sample timing and participant baseline 25(OH)D concentration and/or adiposity [13]. Although vitamin D_3_ is known to quickly rise within 24–48 h after artificial UVB exposure, 25(OH)D is thought to gradually rise and peak 7 to 14 days post exposure [42]. The kinetics of 25(OH)D synthesis, appearance in circulation and sequestration in adipose tissue following sun exposure, however, has not been fully elucidated and may require several repeated exposures under natural conditions [44], perhaps to achieve a certain peak D_3_ concentration. The relatively good status of our participants also may have also contributed. Bogh et al., for example, reported an inverse relationship between baseline 25(OH)D and subsequent increases in 25(OH)D concentration after UVB exposure [50]. Additionally, as previously stated, our participant’s 25(OH)D levels were quite good, and under these conditions, further elevations in circulating 25(OH)D levels become refractory due to enzyme inhibition [51]. Thus, a rise of only of 9.8 nmol vitamin D_3_ is simply not enough to drive product levels higher. Furthermore, adiposity in general was relatively high in some of our participants and could have diminished the appearance of 25(OH)D in circulation. This may be related to adipose tissue sequestration of D_3_ and 25(OH)D following synthesis [52,53].

While to our knowledge this is the first study to evaluate the effect of a single bout of sensible sun exposure on vitamin D_3_ concentration using sunlight exposure for a carefully monitored duration, the study is not without limitations. Limitations include the relatively small sample size, variable characteristics between the older and younger cohorts (the older cohort had more women), inclusion of participants with optimal baseline serum 25(OH)D, inability to study subjects all on the same exposure day and time span between measurements in the older and younger cohort. The later points, however, are an inherent reality of “sensible sun exposure” guidelines. Our exclusionary criteria limited the number of qualified participants—particularly in the older cohort—combined with the unpredictable number of warm, cloudless days and a limited sample size. The dependence on accurate self-reporting and knowledge of dietary vitamin D intake, supplement usage and sun exposure may have also allowed for inclusion of participants with optimal serum 25(OH)D that may have accounted for several non-responders. Additionally, more frequent sampling of D_3_ over the first 72 h (e.g., every 6 to 12 h), a longer follow-up period for 25(OH)D sampling, serial exposures (i.e., three consecutive days) and assessment of age-related thickness differences in the epidermis and/or dermis could be employed in future studies to fully capture the relationship between peak D_3_, serum 25(OHD concentration [42] and ageing to confirm a rise in 25(OH)D relative to the rise in vitamin D_3_ following sun exposure. Employment of non-invasive procedures including optical coherence tomography, two-photon microscopy [10] or confocal laser scanning microscopy [11] would be particularly important to better understand the effect of age-related epidermal thickness changes in relation to vitamin D_3_ production following controlled sunlight exposure.

## 5. Conclusions

A single 30-min bout of sun exposure during late spring at close to solar noon was sufficient to observe an increase in vitamin D_3_ concentration within 24 to 48 h in a cohort of younger and older adults as has been previously observed in the photobiology laboratory with a measured dose of UVB radiation. The response, however, was ~25 to 30% of that observed with delivery of 1 MED in the photobiology laboratory. This study is one of the first to evaluate and practically apply UVB exposure from natural sunlight to examine the effect of subcutaneous vitamin D_3_ synthesis in younger and older adults. Regression modeling of the appearance of D_3_ in circulation suggested that age accounted for approximately 20 percent of the variance in D_3_ production from baseline to peak and revealed a 13% decrease in D_3_ production with every decade of life. Increases in serum 25(OH)D, however, were not observed at 3 days post-exposure in our younger cohort or at 7 days postexposure in our older cohort. Additional research is needed to better formulate sensible sun exposure guidelines that optimize vitamin D status but avert skin damage, which can lead to skin cancers and other sun associated problems [54]. Additional research is needed to determine the influence of age on timing of peak D_3_ concentration, investigate the kinetics of in vivo D_3_ adipose tissue sequestration to explain the relationship between cutaneous D_3_ production and 25(OH)D response and better understand the specific limitations of ageing (which potentially include reduced dermal and epidermal thickness) on cutaneous vitamin D production.

## Figures and Tables

**Table 1:** Participant Characteristics.

	Total Group	Younger Adults	Older Adults	*P* *
Sex (M/F)	11/19	9/9	2/10	—
Age (years)	38.4 ± 10.4 (20–69)	25.1 ± 4.8 (20–37)	58.3 ± 5.1 (50–69)	0.0001 *
Skin Type (II/III)	3/27	3/15	0/12	—
Hair (Bl/Br/R)	7/21/2	5/12/1	2/9/1	—
Mass (kg)	71.2 ± 15.2 (50.1–106.1	71.8 ± 13.4 (54.2–102.1)	70.4 ± 18.2 (50.1–106)	0.816
Height (cm)	170.4 ± 7.9 (156.5–186.0)	173.3 ± 7.4 (156.5–186)	166.2 ± 6.8 (159.0–179)	0.013 *
BMI (kg/m^2^)	24.4 ± 4.5 (18.7–39.4)	23.8 ± 3.2 (20.0–30.0)	25.4 ± 6.1 (18.7–39.4)	0.34
Body Fat (%)	30.3 ± 10.3 (11.9–54.0)	28.8 ± 9.4 11.9–47.7)	32.5 ± 11.5 (12.7–54.0)	0.35
Vitamin D Intake (IU)	187 ± 182 (20–828)	183.5 ± 167.4 (20–828)	151.3 ± 96.2 (64–385)	0.64
Sun Exposure (h/month)	14.5 ± 15.2 (0–60)	13.4 ± 17.4 (0–60)	16.2 ± 16.9 (2–60)	0.63
Baseline Serum D_3_ (nmol/L)	15.0 ± 19.5 (3.7–110.1)	18.0 ± 24.2 (3.7–110.1)	10.7 ± 8.2 (3.7–30.0)	0.33
Baseline Serum D_2_ (nmol/L)	<3.7	<3.7	<3.7	—
Baseline Serum 25(OH)D (nmol/L)	84.9 ± 26.0 (42.4–166.5)	86.6 ± 6.3 (56.7–166.5)	82.6 ± 7.5 (42.4–123.5)	0.69

Data are mean ± SD with ranges shown in parentheses. Bl, blond; Br, brown; R, red BMI, body mass index. * Difference between older and younger cohorts via Independent Samples t-tests.

## Data Availability

Data available upon reasonable request.
